# *Lactobacillus casei* TH14 and Hydrolysable Tannin as Corn Silage Additives Modify In Vitro Rumen Fermentation and Reduce Methane Production

**DOI:** 10.3390/ani16162447

**Published:** 2026-08-07

**Authors:** Rittikeard Prachumchai, Anusorn Cherdthong

**Affiliations:** 1 Department of Agricultural Technology, Faculty of Science and Technology, Thammasat University, Pathum Thani 12120, Thailand; ritprach@tu.ac.th; 2 Department of Animal Science, Faculty of Agriculture, Khon Kaen University, Khon Kaen 40002, Thailand

**Keywords:** chestnut tannin, corn silage, in vitro rumen fermentation, lactic acid bacteria, volatile fatty acids, methane production

## Abstract

This study evaluated *L. casei* TH14 (TH14) and two levels of hydrolysable tannin (HT; sweet chestnut, *Castanea sativa*) as corn ensiling additives, hypothesising that their combination would improve fermentation quality and reduce in vitro CH_4_. Whole-plant corn was ensiled for 21 days in a completely randomised design (seven treatments, four replicates) combining Molasses, TH14, and HT at 20 or 40 g/kg DM. Silage pH was significantly affected by treatment (*p* < 0.01), with T4 reaching the lowest final pH (3.83), whereas the primary nutrients (DM, OM, CP, and NDF) were unchanged (*p* > 0.05). Hydrolysable tannin reduced ruminal NH_3_-N at 24 h (11.12 vs. 13.06 mg/dL; *p* < 0.05) and, at 40 g/kg DM, lowered CH_4_ by 50.0% at 24 h (2.45 vs. 4.90 mL/g DM; *p* < 0.01) in a dose-dependent manner, accompanied by a higher propionate proportion and a lower acetate-to-propionate ratio (*p* < 0.01). Independent of tannin, TH14 reduced CH_4_ by 12.7% (*p* < 0.05). Digestibility was transiently reduced at 24 h but unaffected at 48 h (*p* > 0.05). Corn silage with HT at 40 g/kg DM, alone or combined with TH14, is a promising strategy to mitigate CH_4_ without impairing nutrient digestibility.

## Simple Summary

Corn silage is a critical fermented feed for ruminant livestock in tropical regions, but silage fermentation quality and enteric methane (CH_4_) emissions from fed animals remain key challenges. This study evaluated the combined effects of *Lactobacillus casei* TH14, a native Thai lactic acid bacterium, and Tanno-SAN P, a hydrolysable tannin extract from chestnut (*Castanea sativa*), added to whole-plant corn silage on fermentation characteristics (pH and NH_3_-N), in vitro rumen fermentation, and CH_4_ production. Seven treatment groups were compared over 21 days of ensiling, followed by in vitro incubation with buffered rumen fluid. Hydrolysable tannin, particularly at 40 g/kg dry matter, reduced ammonia nitrogen (NH_3_-N) and in vitro CH_4_ production and increased propionate proportion, while nutrient digestibility was maintained after 48 h. Hydrolysable tannin was the main driver of these effects, whereas *L. casei* TH14 made a smaller independent contribution, and combining the two additives did not produce effects beyond those of tannin alone. These findings suggest that hydrolysable tannin, alone or with *L. casei* TH14, may help improve corn silage use and reduce in vitro CH_4_ production. Further animal studies are needed before field application can be recommended.

## 1. Introduction

Ruminant livestock play an indispensable role in global food security, providing approximately one-third of human dietary protein intake and supporting the livelihoods of more than one billion people worldwide [1]. However, the enteric fermentation processes that characterise ruminant digestion generate substantial quantities of methane (CH_4_), a potent greenhouse gas with a global warming potential approximately 28 times that of CO_2_ over a 100-year horizon. Enteric fermentation in ruminants contributes to approximately 14–18% of global anthropogenic CH_4_ emissions, making enteric fermentation an important target for greenhouse gas mitigation strategies compatible with sustained animal productivity [1,2]. In tropical regions such as Southeast Asia, where the ruminant population is expanding to meet growing protein demands, identifying locally applicable, cost-effective feed additives that simultaneously improve silage quality and reduce enteric CH_4_ production is a pressing research priority. Importantly, an effective additive should not only mitigate CH_4_ but also safeguard silage fermentation quality, including rapid acidification, protein preservation, and dry-matter recovery, which together determine nutritive value [3]. At the rumen level, redirecting fermentation toward propionate is especially desirable, as propionogenesis provides an alternative hydrogen sink that competes with methanogenesis while supplying glucogenic precursors to the host [4].

Among biological additives for silage, lactic acid bacteria (LAB) inoculants have been extensively evaluated for their ability to accelerate acidification, suppress undesirable microorganisms, and preserve forage dry matter and protein fractions during ensiling [5]. Molasses, a readily fermentable carbohydrate source rich in water-soluble sugars, is commonly co-applied with LAB inoculants as a substrate to stimulate lactic acid fermentation and accelerate acidification during ensiling. *Lactobacillus casei* TH14, a homofermentative LAB isolated from sweet corn stover silage in Thailand, has been characterised by high lactic acid production, low pH tolerance, and good adaptability under tropical conditions [6]. These traits make *L. casei* TH14 a robust, locally sourced inoculant well suited to tropical ensiling, where rapid pH decline is critical for limiting proteolysis and nutrient loss [7]. Khota et al. [8] demonstrated that *L. casei* TH14 inoculation improved fermentation quality of tropical grass silages, inhibiting proteolysis and reducing crude protein degradation. In sorghum silage, *L. casei* TH14 supplementation significantly lowered the ratio of in vitro CH_4_ production to total gas production compared with uninoculated controls, suggesting an indirect CH_4_-mitigating effect through improved silage fermentation and altered substrate availability for rumen microbes [9]. More recently, Suntara et al. [10] reported that microbial additives modulated volatile fatty acid (VFA) profiles and in vitro digestibility in corn silage, underscoring the potential of LAB inoculants to improve both silage quality and rumen fermentation efficiency. Nevertheless, it remains unclear whether *L. casei* TH14 applied to whole-plant corn silage can consistently reduce proteolysis and modulate post-fermentation rumen CH_4_ production, particularly when combined with anti-methanogenic plant bioactives.

Hydrolysable tannin (HT) is a class of plant secondary metabolites, containing polyesters of gallic or ellagic acid esterified to polyols, with well-documented anti-methanogenic and anti-proteolytic properties in ruminant nutrition. Unlike condensed tannins, which can form stable complexes with proteins and fibre at high inclusion levels, hydrolysable tannin is more readily hydrolysed in the rumen. Its phenolic products, including gallic acid and pyrogallol, may suppress methanogenesis with less risk of reducing nutrient digestibility [11,12,13]. Meta-analytic evidence from Jayanegara et al. [14] showed that the dietary tannin level was negatively correlated with CH_4_ production across in vitro and in vivo experiments, indicating a dose-dependent response. Nevertheless, the effects of tannins are dose-dependent, and excessive inclusion may depress fibre digestibility, feed intake, and fibrolytic microbial activity [15]; defining an effective yet non-detrimental dose is therefore essential. Chestnut (*Castanea sativa*) HT has shown particular promise for reducing NH_3_-N and CH_4_ in alfalfa silage: Chen et al. [16] reported that tannic acid added to alfalfa silage at 20–40 g/kg DM decreased in vitro CH_4_ production by 10.3–12.4% and improved microbial protein synthesis without impairing rumen fermentation, while Chen et al. [17] demonstrated that combined hydrolysable and condensed tannins in alfalfa silage further reduced CH_4_ and modulated rumen microbiota. In a sheep feeding study, Deaville et al. [12] also demonstrated that chestnut tannin silage shifted N excretion from urinary to faecal pathways, indicating reduced ruminal proteolysis and improved N retention. Most of this evidence, however, derives from alfalfa silage, and comparatively few studies have examined hydrolysable tannin in corn or other cereal silages [18], underscoring the need for the present work. Accordingly, two inclusion levels (20 and 40 g/kg DM) were chosen to capture a dose–response while remaining within a safe and practical range: the lower level reflects concentrations previously effective in reducing CH_4_ from tannin-treated silage, whereas the higher level stays below the threshold (50 g/kg DM) at which tannins begin to compromise intake and fibre digestibility [15]. Despite these encouraging findings, the optimal inclusion level of HT in corn silage, and its interaction with a LAB inoculant such as *L. casei* TH14, has not been examined in the context of tropical corn-based production systems.

Mechanistically, the two additives may act complementarily: *L. casei* TH14 drives rapid lactic acid production and pH decline that preserve dry matter and protein during ensiling, whereas hydrolysable tannin binds protein reversibly to limit in-silo proteolysis and shifts rumen fermentation toward propionate [19,20]. To the authors’ knowledge, the combined effect of *L. casei* TH14 and two inclusion levels of sweet chestnut hydrolysable tannin on corn silage fermentation dynamics across multiple ensiling days, subsequent in vitro rumen fermentation characteristics, nutrient digestibility, and CH_4_ production has not previously been reported. On this basis, it was hypothesised that *L. casei* TH14 and HT would improve silage acidification, reduce protein degradation, and lower in vitro CH_4_ production. We further expected these effects to be associated with changes in rumen fermentation patterns, particularly greater propionate formation, without reducing 48 h nutrient digestibility. The objectives of this study were (1) to evaluate the effects of *L. casei* TH14 and HT supplementation on the chemical composition, pH, and NH_3_-N concentration of corn silage on days 0, 7, 14, and 21 and (2) to determine the effects of the resulting day-21 silages on in vitro rumen gas production kinetics, fermentation characteristics, nutrient digestibility, and CH_4_ production.

## 2. Materials and Methods

### 2.1. Ethics Statement

Rumen fluid was collected from three non-fistulated Thai Brahman cows (*Bos indicus*) maintained at the Faculty of Science and Technology, Thammasat University, Thailand; rumen fluid collection is described in Section 2.4. All experimental procedures involving animals were approved by the Institutional Animal Care and Use Committee of Thammasat University (approval number: IACUC-TU No. 003/2026) and conducted in accordance with the guidelines for the care and use of experimental animals.

### 2.2. Experimental Design and Treatments

Seven treatments were arranged in a completely randomised design (CRD) with four replicates per treatment. Whole-plant corn (*Zea mays* L.) was harvested at 31.50% dry matter (DM) and chopped to a 2–3 cm theoretical length of cut. Treatments were as follows: T1 = Control (no additive); T2 = Molasses (20 g/kg fresh weight); T3 = Molasses + *L. casei* TH14 (10^5^ cfu/g fresh matter); T4 = Molasses + hydrolysable tannin at 20 g/kg DM (Low HT); T5 = Molasses + hydrolysable tannin at 40 g/kg DM (High HT); T6 = Molasses + *L. casei* TH14 + hydrolysable tannin at 20 g/kg DM; T7 = Molasses + *L. casei* TH14 + hydrolysable tannin at 40 g/kg DM. The HT inclusion rate was calculated based on the dry matter of fresh chopped corn before ensiling.

The hydrolysable tannin source was Tanno-SAN P (Sanluc International nv, Gent, Belgium), a commercial sweet chestnut (*Castanea sativa*) extract containing predominantly ellagitannins and gallotannins (total polyphenols >50%; GMP + FSA Assured). The tannin product used in this study was supplied by Vet Innovation Technology Company Limited. Molasses (20 g/kg FM) and the hydrolysable tannin (20 or 40 g/kg DM) were dissolved in distilled water and sprayed uniformly onto the chopped corn to ensure even distribution. The Control and all treatments received an equal volume of distilled water so that added moisture was similar across treatments.

The *L. casei* TH14 inoculant was prepared by culturing the strain overnight in de Man, Rogosa, and Sharpe (MRS) broth (Difco Laboratories, Detroit, MI, USA) at 37 °C. The optical density of the suspension at 620 nm was adjusted to 0.42 using sterile 0.85% NaCl solution to achieve an inoculation rate of 1 × 10^5^ cfu/g fresh matter, following the method described by Pholsen et al. [6]. Approximately 500 g of treated fresh corn was packed into polyethylene-laminated nylon bags and sealed under vacuum to exclude oxygen. All silos were stored at ambient temperature (~28–35 °C) and opened on day 21 for evaluation.

### 2.3. Silage Sampling and Chemical Analysis

Silage samples were collected destructively on days 0, 7, 14, and 21 of ensiling. Silage pH was determined by homogenising 20 g of fresh silage in 250 mL of distilled water and soaking it at 4 °C for 2 h; the extract was then filtered and measured with a calibrated glass electrode [6]. Separate silo replicates were prepared for each treatment and ensiling day; therefore, each silo was opened only once. Sub-samples were dried at 60 °C for 48 h, ground to pass through a 1 mm screen, and subjected to chemical analysis. DM (method 930.15) and ash (method 942.05) were determined following AOAC [21], with organic matter (OM) calculated by difference. Crude protein (CP) was determined by the Kjeldahl method (method 976.05; N × 6.25; AOAC [21]). Neutral detergent fibre (NDF) and acid detergent fibre (ADF) were sequentially determined using heat-stable α-amylase and sodium sulphite with the ANKOM 200 Fiber Analyser (ANKOM Technology, Macedon, NY, USA) according to Van Soest et al. [22] and expressed on an ash-inclusive basis. Gross energy (GE) was determined by bomb calorimetry (Parr Instruments, Moline, IL, USA). Ammonia nitrogen was determined colourimetrically using the phenol–hypochlorite method [23]. For NH_3_-N determination, 20 g of fresh silage was homogenised in 250 mL of distilled water, and the mixture was refrigerated at 4 °C for 2 h and filtered; the filtrate was analysed by the phenol–hypochlorite method [23], and NH_3_-N was expressed as mg/dL of silage extract.

### 2.4. Rumen Fluid Preparation and In Vitro Incubation

Rumen fluid was collected from the same three non-fistulated Thai Brahman donor cows described in Section 2.1 that were fed rice straw ad libitum supplemented with concentrate at 1% body weight (75% TDN, 14% CP). Rumen contents were collected via oral stomach tube before the morning feeding: a flexible polyethylene tube (stomach tube) was inserted transorally through a speculum into the rumen, and rumen fluid was withdrawn by gentle vacuum suction using a hand-operated vacuum pump into a pre-warmed collection vessel. The collected fluid was immediately strained through four layers of cheesecloth and transported to the laboratory in pre-warmed insulated flasks maintained under continuous CO_2_ flushing. To minimise salivary contamination, the tube was inserted through a rigid speculum and the first portion of aspirate was discarded. Rumen fluid pH measured immediately after collection was 6.8–6.9, within the normal range for pre-feeding ruminal fluid, indicating negligible salivary contamination. Rumen fluid was pooled from three donors and maintained at 39 °C under continuous CO_2_ flushing until use. Because rumen fluid was pooled before incubation, the donor animal was not used as an experimental unit in the in vitro analysis. Buffered rumen fluid was prepared by mixing rumen fluid with CO_2_-saturated McDougall’s artificial saliva [24] at a ratio of 1:2 (*v*/*v*), with pH adjusted to 6.8 ± 0.1.

The fermentation substrate for in vitro incubation was a mixture of day-21 corn silage (0.3 g DM) and concentrate diet (0.2 g DM), totalling 0.5 g DM per incubation unit, to reflect a practical roughage-to-concentrate ratio used in Thai Brahman feeding systems. The ingredients and chemical composition of the concentrate diet are presented in Table 1. The concentrate was ground to pass through a 1 mm screen prior to weighing. Within each of the three independent runs, three independent sets of 50 mL serum vials were prepared from the same batch of buffered rumen fluid: Set A for cumulative gas production (four vials per treatment + four blank vials containing buffered rumen fluid only, without substrate); Set B for rumen fermentation characteristics (pH, NH_3_-N, VFA, CH_4_, and protozoa; four vials per treatment per time point × two time points: 24 and 48 h); and Set C for in vitro nutrient degradability (four vials per treatment per time point × two time points: 24 and 48 h). Blank vials were included in each set to correct for background gas production and microbial residue from the rumen inoculum. All gas and fermentation values reported were blank-corrected.

Samples of the total substrate (0.5 g DM, consisting of 0.3 g of day-21 silage and 0.2 g of concentrate as described above) were weighed into 50 mL serum vials and incubated with 40 mL of buffered rumen fluid at 39 °C using the in vitro gas production technique of Suriyapha et al. [25]. Gas volume was measured manually by reading the displacement of a calibrated 20 mL graduated glass syringe at 0.5, 1, 2, 4, 6, 8, 12, 18, 24, 36, 48, 72, and 96 h post-incubation, following the technique of Menke and Steingass [26]. Cumulative gas production data were fitted to the logistic model of Schofield [27]: *V*(*t*) = *b* × [1 − exp^(−c(*t*−*L*))^], where *b* is the final asymptotic gas volume corresponding to the fully digested substrate (mL/0.5 g DM), *t* is the incubation time (h), c is the rate constant (h^−1^), and *L* is the lag term phase (h). Model parameters were estimated by non-linear least squares regression.

### 2.5. Rumen Fermentation Characteristics

At 24 and 48 h of incubation, vials were removed from the incubator and contents collected. Rumen pH was measured using a calibrated glass electrode. Samples were centrifuged at 3000× *g* for 15 min at 4 °C, and the supernatant was used for analysis of NH_3_-N and VFA; a separate 1 mL aliquot of fresh rumen fluid was retained prior to centrifugation for protozoa enumeration. Ruminal NH_3_-N was determined by the phenol–hypochlorite colorimetric method [23]. Protozoa were enumerated using the methyl-green–formalin–saline (MGFS) staining method described by Galyean [28]. Briefly, a 1 mL aliquot of fresh rumen fluid was fixed in the MGFS solution (1:1 *v*/*v*), and total protozoa were counted under a light microscope using a haemocytometer at ×100 magnification. Results were expressed as ×10^6^ cells/mL.

Volatile fatty acid concentrations (acetate, propionate, and butyrate) were determined by high-performance liquid chromatography (HPLC) following the method of Samuel et al. [29]. Briefly, supernatant samples were deproteinised with metaphosphoric acid (25%, *w*/*v*) and centrifuged at 12,000× *g* for 15 min at 4 °C. The clarified supernatant was filtered through a 0.22 μm membrane filter and injected onto an Aminex HPX-87H organic acid column (Bio-Rad Laboratories, Hercules, CA, USA) at 60 °C, using 0.005 M H_2_SO_4_ as the mobile phase at a flow rate of 0.6 mL/min. VFAs were detected by a refractive index (RI) detector. Crotonic acid was used as an internal standard. Total VFA was expressed as mmol/L and individual VFAs as mol/100 mol total VFA.

### 2.6. Methane Production

Methane production was measured directly by gas chromatography at 24 and 48 h post-incubation in a dedicated set of vials incubated in parallel with, and under identical conditions to, the gas-kinetics vials. At each time point, a 10 mL gas sample was extracted from the headspace of the serum vial using a gas-tight syringe and immediately injected into a gas chromatograph equipped with a flame ionisation detector (Shimadzu Corporation, Kyoto, Japan). The GC column was a stainless steel packed column (Porapak Q, 80/100 mesh; 2 m × 3 mm) with nitrogen as the carrier gas at a flow rate of 30 mL/min; column, injector, and detector temperatures were set at 50, 100, and 150 °C, respectively. Methane concentration was quantified against a certified CH_4_ standard gas and expressed as mL/g DM incubated. These time points reflect the mean ruminal retention time (24 h) and near-complete fermentation (48 h), while total gas was recorded to 96 h to capture full kinetics.

### 2.7. In Vitro Nutrient Digestibility

In vitro dry matter digestibility (IVDMD), organic matter digestibility (IVOMD), neutral detergent fibre digestibility (IVNDF), and true digestibility (IVTD) were determined at 24 and 48 h of incubation using the two-stage technique of Tilley and Terry [30], adapted for use with serum vial incubation residues.

At the end of each incubation period, vial contents were quantitatively transferred onto pre-weighed sintered glass crucibles (porosity 2; Schott Duran, Mainz, Germany) and filtered under vacuum. The residues were washed three times with a neutral detergent solution [22] to remove soluble fermentation products and microbial biomass, then rinsed twice with distilled water and once with acetone. Subsequently, residues were filtered, dried at 105 °C for 24 h, and weighed to determine the undigested DM residue. A blank (buffered rumen fluid without a substrate) and a reference standard (meadow hay of known digestibility) were included in each incubation run to correct for microbial residue and to validate analytical accuracy.

IVDMD was calculated as apparent DM disappearance after incubation, whereas IVTD was calculated after neutral detergent extraction of residues to account for microbial contamination. IVOMD and IVNDF were calculated as the disappearance of initial OM and NDF, respectively. All values are expressed on a % DM basis.

### 2.8. Statistical Analysis

Data were analysed using the MIXED procedure of SAS (version 9.4; SAS Institute, Cary, NC, USA). For the silage data in Table 2, treatment, ensiling day, and treatment × day interaction were included as fixed effects. Silo replicate was included as the random effect. Because separate silos were opened at each ensiling day, day was treated as a fixed factor rather than as a repeated measure on the same silo. A separate set of silos was prepared for each opening day; four replicate silos per treatment were destructively sampled at each of days 0, 7, 14, and 21, giving a total of 112 silos (7 treatments × 4 days × 4 replicates).

For the in vitro data in Table 3, Table 4, Table 5 and Table 6, treatment was included as the fixed effect, and incubation run was included as the random effect. The in vitro incubation was conducted in three independent runs on different days. Within each run, rumen fluid from the three donor cows was pooled before incubation; therefore, the donor animal was not used as an experimental unit. The incubation vial was considered the observational unit within each run.

Least squares means were separated using Tukey’s honest significant difference (HSD) test at *p* < 0.05. Four a priori planned comparisons were evaluated using CONTRAST statements: Contrast 1 = Control (T1) vs. mean of all Molasses-based treatments (T2–T7); Contrast 2 = Molasses alone (T2) vs. Molasses + *L. casei* TH14 (T3); Contrast 3 = treatments without HT (T2, T3) vs. treatments with HT (T4, T5, T6, T7); Contrast 4 = Low HT treatments (T4, T6) vs. High HT treatments (T5, T7). These contrasts were specified as planned comparisons to address a priori hypotheses about additive effects, and statistical significance was declared at *p* < 0.05.

## 3. Results

### 3.1. Chemical Composition and Fermentation Profile of Corn Silage

The chemical composition and fermentation profile of all treatments across ensiling days are presented in Table 2. Crude protein and NDF were unaffected by treatment, ensiling day, or their interaction (*p* > 0.05). Gross energy showed no interaction (*p* = 0.3587) but was affected by treatment (*p* < 0.01), with the Control (T1) having the lowest value (16.81 vs. 16.93–17.16 MJ/kg DM), and by ensiling day (*p* < 0.01). For DM, OM, ADF, and pH, the treatment × day interaction was significant (*p* < 0.01), indicating that the additive effects varied with ensiling stage: DM and ADF changed as ensiling progressed, silage pH declined from a mean of 5.05 on day 0 to 3.99 on day 21, and treatment differed on day 0 (range: 4.61–5.39) but converged to uniformly low values by day 21 (3.92–4.12). Ammonia nitrogen showed no significant interaction (*p* = 0.09) but differed among treatments (*p* < 0.01) and increased with ensiling time (0.62 to 1.98 mg/dL; *p* < 0.01): the Control retained the highest NH_3_-N (2.11 mg/dL), whereas the high-tannin treatments (T5–T7) had the lowest NH_3_-N (0.98–1.30 mg/dL), consistent with the tannin-mediated protection of silage protein.

### 3.2. Gas Production Kinetics and Cumulative Gas Production

**Figure 1:**
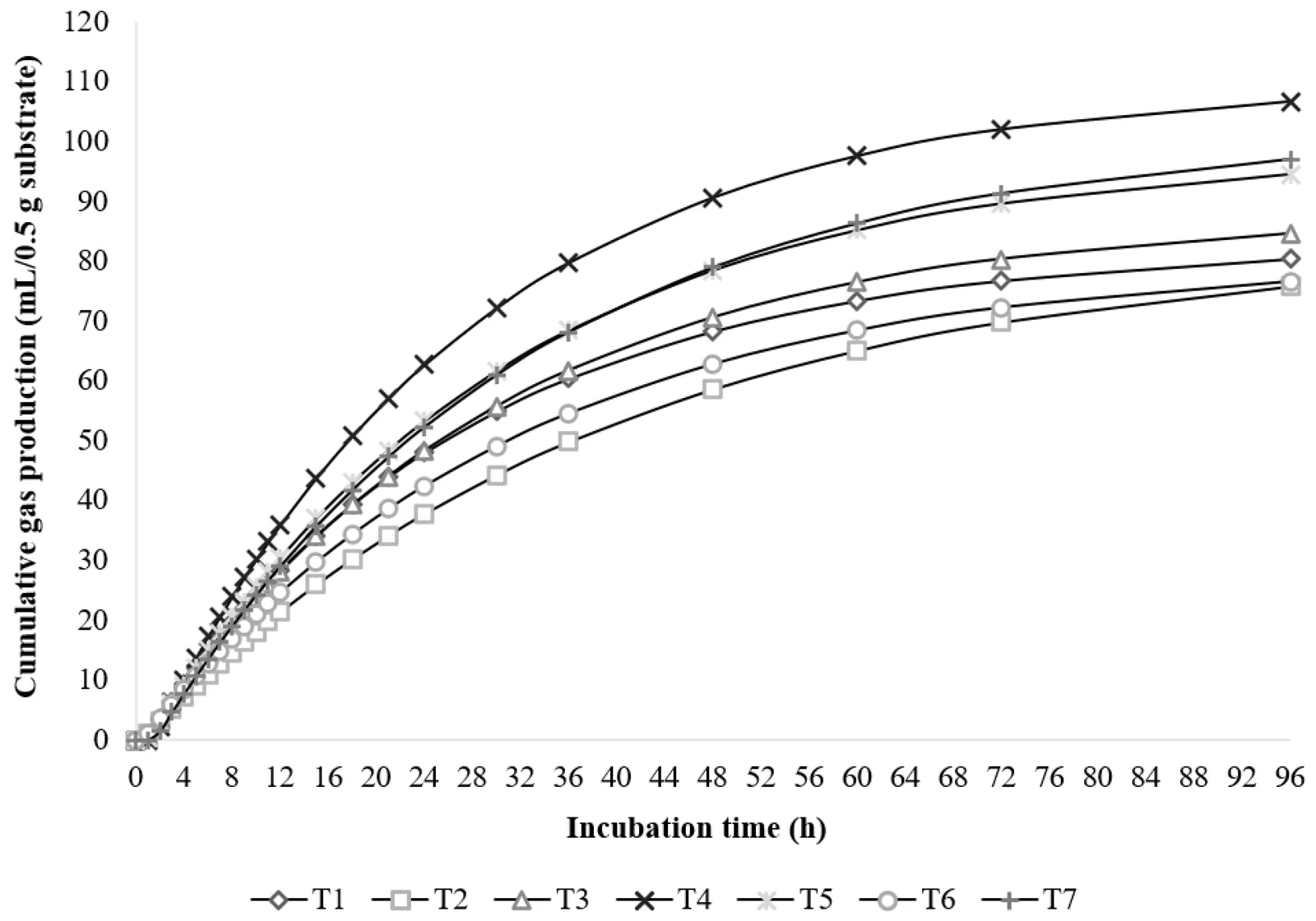
Effects of corn silages treated with *Lactobacillus casei* TH14 and hydrolysable tannin on cumulative gas production during incubation.

Gas production kinetics and cumulative gas production are presented in Table 3 and Figure 1. The maximum potential gas production (*b*) differed among treatments (*p* < 0.01), being highest in T4 (Molasses + Low HT; 109.95 mL/0.5 g DM), whereas the rate constant (c) and lag phase (*L*) were unaffected (*p* > 0.05). Cumulative gas production also differed among treatments at 12, 24, 48, and 96 h (*p* < 0.05). Among the planned contrasts, hydrolysable tannin inclusion (Contrast 3) increased cumulative gas at all time points (52.66 vs. 43.07 mL/0.5 g DM at 24 h) and the b value (97.68 vs. 85.43 mL/0.5 g DM; *p* < 0.05), whereas the comparisons of Control versus Molasses (Contrast 1), Molasses versus Molasses plus *L. casei* TH14 (Contrast 2), and low versus high tannin dose (Contrast 4) did not significantly affect gas production (*p* > 0.05).

### 3.3. Ruminal pH, Ammonia Nitrogen, Methane Production, and Protozoa Population

Ruminal fermentation parameters are presented in Table 4. Ruminal pH remained within the normal range (6.73–6.92) and was unaffected by treatment at 24 or 48 h (*p* > 0.05). Ruminal NH_3_-N was reduced by treatment at 24 h (*p* < 0.05), with T5 showing the lowest value (10.43 vs. 11.21–13.40 mg/dL in the other treatments); the contrast for hydrolysable tannin inclusion (Contrast 3) confirmed lower NH_3_-N with HT (11.12 vs. 13.06 mg/dL; *p* < 0.05). Methane production was reduced by treatment at both 24 and 48 h (*p* < 0.01): at 24 h, the tannin-containing treatments (T4–T7; 2.45–4.05 mL/g DM) produced less CH_4_ than the Control and non-tannin treatments (T1–T3; 4.80–5.50 mL/g DM), and the dose contrast (Contrast 4) confirmed a dose-dependent reduction (high vs. low HT: 2.58 vs. 3.85 mL/g DM; *p* < 0.05). Protozoa counts were unaffected by treatment (*p* > 0.05), although they tended to decrease numerically with increasing tannin inclusion.

### 3.4. In Vitro Nutrient Digestibility

In vitro nutrient digestibility is presented in Table 5. Overall, IVDMD, IVOMD, IVNDF, and IVTD were not significantly affected by treatment at 24 h (IVDMD: *p* = 0.069; IVOMD: *p* = 0.063; IVNDF: *p* = 0.170; IVTD: *p* = 0.069) or at 48 h (all *p* > 0.05). Nevertheless, the planned contrasts revealed transient effects at 24 h: hydrolysable tannin inclusion (Contrast 3) slightly reduced IVTD (83.55 vs. 85.23%; *p* < 0.05), and the high tannin dose (Contrast 4) lowered IVDMD (54.11 vs. 56.71%; a 4.6% reduction) and IVOMD (56.54 vs. 59.39%; a 4.8% reduction) relative to the low dose (*p* < 0.05). No significant differences were detected among any contrasts at 48 h, indicating that early digestibility effects were transient.

### 3.5. Volatile Fatty Acid Concentrations

Volatile fatty acid concentrations are presented in Table 6. Total VFA was reduced by treatment at both 24 and 48 h (*p* < 0.05), with the total VFA of high-tannin treatments (T5, T7) being the lowest at 24 h. Propionate proportion and the acetate-to-propionate (C2:C3) ratio were both affected at 24 and 48 h (*p* < 0.01): the tannin treatments raised propionate above the Control (15.04 mol/100 mol at 24 h) and correspondingly lowered the C2:C3 ratio, which was the highest in the Control (4.89 at 24 h; 4.96 at 48 h) and the lowest in the *L. casei* TH14-containing treatments (T3, T6, T7; 3.68–3.89 at 48 h). The planned contrasts confirmed these effects: *L. casei* TH14 increased propionate at 48 h (Contrast 2; 19.05 vs. 17.00 mol/100 mol; *p* < 0.05), hydrolysable tannin increased propionate at 24 h and reduced total VFA at both times (Contrast 3; *p* < 0.05), and the high tannin dose further lowered total VFA at 24 h (Contrast 4; 42.00 vs. 46.53 mmol/L; *p* < 0.05).

## 4. Discussion

The present study tested the hypothesis that *L. casei* TH14 combined with hydrolysable tannin would improve corn silage fermentation and reduce in vitro rumen methane production. This hypothesis was largely supported: hydrolysable tannin, particularly at 40 g/kg DM, lowered silage NH_3_-N and reduced CH_4_ production while increasing propionate, whereas *L. casei* TH14 contributed a smaller, independent reduction in CH_4_. However, the combination did not act synergistically, and 48 h nutrient digestibility was preserved.

### 4.1. Chemical Composition and Fermentation Profile of Corn Silage

The progressive decline in silage pH from day 0 to day 21 indicated adequate acidification. Final pH values were below the 4.2 threshold commonly associated with acceptable preservation of whole-plant corn silage [8]. The significant treatment × day interaction for pH indicates that the combination of *L. casei* TH14 and hydrolysable tannin modulated the rate of acidification rather than merely the final equilibrium pH. Notably, T6 showed the lowest pH on day 0. This early difference likely reflects the initial additive mixture rather than lactic acid production, as the day-0 reading was measured before substantial ensiling had occurred. Consistently, T7 showed no comparable early decline despite its higher tannin dose, indicating that these day-0 differences arose from the additive mixture rather than from fermentation. The lower pH observed after ensiling is consistent with Cherdthong et al. [31], who reported that *L. casei* TH14 with Molasses accelerated pH decline in rice straw silage. Similarly, So et al. [32] demonstrated that *L. casei* TH14 addition to sugarcane bagasse silage reduced pH within the first week of ensiling, reflecting efficient early-stage fermentation.

The DM content increased significantly with ensiling time (*p* < 0.01); the slightly lower DM on day 0 relative to the fresh chopped corn (31.50%) reflects the moisture added with the additive solutions, after which DM rose as fermentation progressed [6]. The lack of a significant treatment effect on CP content (*p* = 0.313) suggests that the additives did not alter total crude protein concentration during ensiling. However, hydrolysable tannin may have affected protein fractions through tannin–protein complexation. Hydrolysable tannin from sweet chestnut (Castanea sativa), as used in the present study (Tanno-SAN P), is known to form reversible complexes with dietary proteins via hydrogen bonding, thereby protecting soluble protein from microbial degradation during ensiling [11,13]. This protein-protective mechanism is supported by the significantly lower silage NH_3_-N in the high-tannin treatments (T5–T7) compared with the Control (*p* < 0.01), consistent with reduced proteolysis during ensiling. This agrees with Chen et al. [16,17], who reported lower NH_3_-N-to-total-N ratios in tannin-treated alfalfa silage. The relatively high day-21 NH_3_-N in T4 may reflect partial hydrolysis of tannin–protein complexes at the low tannin dose, releasing previously bound nitrogen.

The elevated NH_3_-N in T4 (Molasses + Low HT, 3.15 mg/dL) on day 21, which exceeded that of T1 (2.86 mg/dL), merits further consideration. One possible explanation is that 20 g/kg DM HT was not sufficient to consistently suppress proteolysis during the later stage of ensiling. However, this remains speculative because silage microbial populations were not measured. Furthermore, the mild acidic environment on day 21 (pH 3.83) in T4 may have promoted partial hydrolysis of tannin–protein complexes, releasing previously bound nitrogen back into the fermentation liquor. In contrast, T5 (High HT, 40 g/kg DM) maintained the lowest NH_3_-N across all time points, supporting a dose-dependent protective effect of hydrolysable tannin on silage protein integrity, consistent with observations by Chen et al. [16] using chestnut tannin in alfalfa silage.

### 4.2. Gas Production Kinetics and Cumulative Gas Production

The maximum potential gas production (*b*) was higher in HT-supplemented treatments than in non-HT treatments, and Contrast 3 supported an effect of HT inclusion. This finding is somewhat paradoxical given that tannins are generally considered to reduce fermentation through their antimicrobial and anti-nutritional properties [14]. Hydrolysable tannin differs from condensed tannins in ruminal behaviour. It is more readily hydrolysed and may release phenolic compounds such as gallic acid and ellagic acid [12,13]. At moderate inclusion levels, these compounds may contribute to fermentation, although this was not directly measured in the present study. This substrate-provision effect at 20–40 g/kg DM may explain the elevated b values, as additional fermentable carbon from tannin hydrolysis products increased total gas yield without a corresponding increase in the rate constant (c, *p* = 0.352) or lag phase (*L*, *p* = 0.230). In contrast, Battelli et al. [33] reported that chestnut HT at 2–8% inclusion linearly reduced total gas production during 48 h in vitro incubation with a hay-based substrate, attributing the reduction to impaired dry matter digestibility rather than direct microbial inhibition. The discrepancy with the present findings likely reflects the difference in substrate: corn silage provides a higher proportion of rapidly fermentable non-structural carbohydrates than hay, which may dilute the digestibility-suppressing effect of HT while amplifying the substrate contribution from tannin hydrolysis products. This interpretation is supported by the absence of treatment effects on IVDMD and IVOMD at 48 h, suggesting that HT at 20–40 g/kg DM did not persistently impair substrate utilisation.

The notably high *b* value of T4 (109.95 mL/0.5 g DM) relative to T5 (98.40 mL) and T7 (102.00 mL) warrants attention. T6 (Molasses + *L. casei* TH14 + Low HT), which shares the same HT dose as T4, had a substantially lower b value (80.35 mL), suggesting that the concurrent presence of *L. casei* TH14 suppressed the gas-elevating effect of Low HT. The lower gas production in T6 compared with T4 suggests that *L. casei* TH14 changed the fermentation response to Low HT. The mechanism is unclear because lactic acid, tannin hydrolysis products, and microbial community composition were not measured. The observation that T3 (Molasses + *L. casei* TH14 alone) also failed to increase b beyond the Control range corroborates this interpretation, as *L. casei* TH14-derived lactate-rich end-products may modulate the rumen microbial community toward acetate-and-lactate utilising pathways that do not generate proportionally more gas per unit of substrate degraded [34]. This is consistent with Pongsub et al. [35], who reported no significant effect of *L. casei* TH14 on in vitro gas production kinetics of fermented cassava pulp. One possible explanation is that lactic acid is converted to propionate through the acrylate pathway. Therefore, LAB activity may not always increase gas volume in proportion to substrate fermentation. The lack of significant differences in c among treatments further supports the conclusion that the treatment effects on cumulative gas production were primarily attributable to differences in the quantity of fermentable substrate available, rather than changes in microbial metabolic rate or community composition per se. This distinction has important implications for interpreting gas production kinetics as a proxy for digestibility in tannin-supplemented substrates.

### 4.3. Ruminal pH and Ammonia Nitrogen Concentration

Ruminal pH remained within the physiologically acceptable range for rumen fermentation (6.73–6.92) across all treatments and time points, with no significant treatment effect at either 24 h (*p* = 0.377) or 48 h (*p* = 0.168). This result is consistent with previous in vitro studies using corn silage as a substrate [9,10], where the strong buffering capacity of McDougall’s artificial saliva prevented treatment-induced pH differences. The absence of ruminal pH differences is nutritionally important, as it indicates that HT inclusion at 20–40 g/kg DM did not disrupt the rumen fermentation environment, which could otherwise impair fibre-digesting bacteria sensitive to acidic conditions.

Ruminal NH_3_-N was significantly reduced by HT at 24 h (*p* < 0.05), with T5 recording 10.43 mg/dL versus 12.70 mg/dL for T1, and Contrast 3 suggesting a collective reduction for all HT treatments (11.12 vs. 13.06 mg/dL; *p* < 0.05). This reduction may be related to the ability of hydrolysable tannin to form protein–tannin complexes at near-neutral rumen pH. Such complexes can reduce protein susceptibility to microbial proteolysis and deamination [11,36]. Protein–tannin complexes are mainly formed through hydrogen bonding and hydrophobic interactions. These complexes are relatively stable at rumen pH but may dissociate under acidic abomasal conditions. This process can increase the amount of protein available for post-ruminal digestion [12]. This mechanism is well established across tannin types: Patra and Saxena [11] confirmed in a meta-analysis that dietary tannins consistently lowered ruminal NH_3_-N regardless of tannin class, and a more recent meta-analysis by Brutti et al. [37] confirmed that inclusion of tannins in ruminant diets reduced ruminal N-NH_3_ concentration by a mean of 15% across 44 in vitro and in vivo experiments, with hydrolysable tannin showing comparable efficacy to condensed tannins at equivalent phenolic concentrations. Consistent with this, Prapaiwong et al. [38] reported reduced ruminal ammonia and improved nutrient utilisation with chestnut hydrolysable tannin in crossbred dairy cows. The 15.1% reduction in NH_3_-N for HT treatments in the present study is within this reported range. This supports the biological plausibility of the observed response.

The lack of a significant treatment effect at 48 h (*p* = 0.260) may reflect progressive hydrolysis of tannin–protein complexes during extended in vitro incubation, possibly as cellulolytic and proteolytic bacteria adapt to the tannin-modified environment, gradually releasing bound nitrogen, although this was not measured. This time-dependent response is consistent with the reversible nature of tannin–protein complexes [36]. The NH_3_-N-suppressing effect may be stronger at early incubation times and weaker later as complexes are gradually hydrolysed. The absence of a significant Contrast 2 effect suggests that the reduction in NH_3_-N was mainly associated with HT rather than *L. casei* TH14. This is consistent with the protein-binding capacity of tannins. Cherdthong et al. [31] similarly reported that *L. casei* TH14 supplementation alone did not significantly alter ruminal NH_3_-N in in vitro incubations of rice straw-based substrates, reinforcing the conclusion that LAB inoculants and hydrolysable tannin exert their effects through distinct and largely non-overlapping mechanisms—a distinction with practical significance for formulating combination strategies that maximise silage protein protection.

### 4.4. Methane Production

The most notable finding was the dose-dependent reduction in in vitro CH_4_ production by hydrolysable tannin supplementation. T5 (40 g/kg DM HT) reduced CH_4_ at 24 h by 50.0% relative to the Control (2.45 vs. 4.90 mL/g DM; *p* < 0.01), and T7 (TH14 + 40 g/kg DM HT) produced comparably low CH_4_ (2.70 mL/g DM). This magnitude of reduction substantially exceeds the 8–27% reductions commonly reported for condensed tannins [14,20] and aligns with the higher efficacy reported for hydrolysable tannin at moderate dietary inclusion rates [13]. Contrast 4 confirmed a significant dose–response, with High HT producing less CH_4_ than Low HT at 24 h (2.58 vs. 3.85 mL/g DM; *p* < 0.05).

One possible mechanism is that phenolic compounds released during tannin hydrolysis may suppress methanogenesis. However, this was not directly confirmed because methanogenic archaea and hydrogen concentration were not measured [2,11]. The increase in propionate proportion and the decrease in C2:C3 ratio suggest that hydrogen use may have shifted away from methanogenesis toward propionate formation (Table 6; Contrast 3, *p* < 0.05). Since propionate synthesis is an H_2_-consuming pathway competing directly with CO_2_ reduction to CH_4_ by methanogens, this biochemical shift provides a mechanistically coherent explanation for the observed CH_4_ suppression [4,36].

Contrast 1 demonstrated that Molasses-based treatments collectively reduced CH_4_ compared with the Control (*p* < 0.05), suggesting that readily fermentable carbohydrates from Molasses stimulated propionate-producing bacteria and increased competitive H_2_ utilisation. Contrast 2 further showed that *L. casei* TH14 addition to Molasses reduced CH_4_ at 24 h (4.80 vs. 5.50 mL/g DM; 12.7% reduction; *p* < 0.05), consistent with reports by Cherdthong et al. [31] who attributed this to *L. casei* TH14-derived lactic acid acting as a propionate precursor via the acrylate pathway. However, the magnitude of LAB-only CH_4_ reduction was markedly smaller than that of HT inclusion (12.7% vs. 50.0% for T5), indicating that hydrolysable tannin is the primary driver of CH_4_ mitigation in this system. Thus, although hydrolysable tannin exerted the stronger effect, *L. casei* TH14 independently contributed to CH_4_ reduction and silage acidification, justifying its evaluation as a co-additive.

Protozoa counts were numerically lower in some HT treatments, but the effect was not statistically significant. Therefore, the present data do not support protozoal suppression as a confirmed mechanism of CH_4_ reduction. The well-established endosymbiotic relationship between rumen protozoa and hydrogenotrophic methanogens suggests that reduced protozoal populations could be associated with lower methanogenic activity [39], though this was not confirmed here. The failure to reach statistical significance is likely attributable to high within-treatment variability, and future studies with direct methanogen quantification by qPCR [40] would permit more definitive conclusions. The 50.0% reduction in CH_4_ at 40 g/kg DM is larger than the 10.3–12.4% decrease reported by Chen et al. [16] for tannic acid in alfalfa silage, and exceeds the about 20% mitigation ceiling summarised in the tannin meta-analyses of Min et al. [3] and Piñeiro-Vázquez et al. [41], likely reflecting the higher effective tannin dose and the in vitro batch-culture conditions of the present study. Similarly, Prodanović et al. [42] reported significant reductions in enteric methane emission and yield in mid-lactation cows supplemented with chestnut tannin extract. As microbial populations were not quantified in this study, these mechanistic interpretations remain speculative and require confirmation by direct methanogen and protozoal enumeration.

### 4.5. In Vitro Nutrient Digestibility

In vitro dry matter, organic matter, NDF, and true digestibility were not significantly affected by treatment at 48 h (*p* = 0.346–0.979), suggesting that hydrolysable tannin at 20–40 g/kg DM achieved substantial CH_4_ mitigation without impairing nutrient digestibility. This stands in contrast to condensed tannins, which consistently reduce IVDMD and IVOMD above ~50 g/kg DM through stable protein–fibre cross-links resistant to enzymatic degradation [11,14]. Hydrolysable tannin, being readily hydrolysed in the rumen to simpler phenolic acids, is less likely to form permanent cross-links with cell wall polysaccharides, explaining its more favourable digestibility profile at the concentrations tested [12,13].

The minor but statistically significant reductions in IVTD (Contrast 3) and IVDMD/IVOMD (Contrast 4) at 24 h suggest that, at 40 g/kg DM, HT transiently impaired the initial rate of substrate degradation, likely through the inhibition of cellulolytic bacteria (*Fibrobacter succinogenes*, *Ruminococcus flavefaciens*) sensitive to phenolic compounds. Recovery of full digestibility by 48 h may indicate rapid adaptation of the microbial community once initial tannin effects subsided—a time-dependent phenomenon previously described for polyphenol-containing feeds [36]. The absence of negative digestibility effects at 48 h is practically relevant because it suggests that HT did not persistently reduce substrate degradation in vitro. However, effects on intake, animal productivity, and whole-animal CH_4_ emissions need confirmation in vivo.

### 4.6. Volatile Fatty Acid Concentrations

The significant increase in propionate proportion across all Molasses-based treatments at 24 h, and particularly in T3, T6, and T7 at 48 h, is consistent with a fermentation shift toward gluconeogenic VFA pathways driven by readily fermentable carbohydrates and metabolic H_2_ sinks. This propionate response is consistent with the meta-analytic findings of Yanza et al. [43] and Orzuna-Orzuna et al. [44], who reported systematic increases in ruminal propionate with dietary tannin supplementation. The concurrent increase in propionate proportion and decrease in the C2:C3 ratio are consistent with the propionate-driven redirection of hydrogen away from methanogenesis discussed in Section 4.4.

The reduction in total VFA in T5 and T7 at 24 h (Contrast 4, *p* < 0.05) likely reflects inhibition of some fermentation pathways at High HT concentrations, particularly acetate and butyrate production, while propionate synthesis was relatively spared. The parallel decline in acetate and butyrate—both H_2_-yielding pathways—alongside elevated propionate is consistent with this coordinated redirection of H_2_ metabolism [2,36].

The most pronounced reduction in the C2:C3 ratio at 48 h occurred in the *L. casei* TH14-containing treatments (T3, 3.68; T6, 3.83; T7, 3.89), and Contrast 2 confirmed that *L. casei* TH14 increased propionate at 48 h relative to Molasses alone. This supports a role of *L. casei* TH14 in promoting propionate-oriented fermentation, with lactic acid being converted to propionate via the acrylate pathway by rumen bacteria. Kaewpila et al. [45] likewise reported that *L. casei* TH14 combined with Molasses enhanced lactic acid fermentation, increased propionate, and reduced in vitro CH_4_ intensity in Sesbania grandiflora pod silage.

Together, the VFA data indicate that *L. casei* TH14 and hydrolysable tannin modified rumen fermentation in vitro. The main response was a shift toward a higher propionate proportion and a lower C2:C3 ratio. This occurred without a persistent reduction in 48 h substrate digestibility.

When *L. casei* TH14 and hydrolysable tannin were combined (T6 and T7), the reductions in NH_3_-N and CH_4_ did not exceed those achieved by hydrolysable tannin alone (T4 and T5), indicating that the two additives acted largely additively rather than synergistically. Hydrolysable tannin was the dominant driver of the responses, while the contribution of *L. casei* TH14—although significant for CH_4_ and propionate on its own (Contrast 2)—was not amplified by tannin co-application.

This study has some limitations. Fermentation losses, aerobic stability, the complete organic acid profile (lactic, acetic, propionic, and butyric acids), and microbial counts (lactic acid bacteria, yeasts, and moulds) of the silages were not determined, and ruminal microbial populations were not quantified in the in vitro phase. These parameters would provide a more complete characterisation of both silage fermentation and the underlying rumen microbial responses, and are recommended for future work.

## 5. Conclusions

Under the conditions of this in vitro study, sweet chestnut hydrolysable tannin, either alone or combined with *L. casei* TH14, accelerated silage acidification and significantly lowered silage NH_3_-N, whereas the primary chemical composition (DM, OM, CP, and NDF) remained largely unchanged. At 40 g/kg DM, hydrolysable tannin reduced in vitro CH_4_ production by up to 50.0% and shifted rumen fermentation toward propionate without impairing 48 h nutrient digestibility. Molasses itself contributed to CH_4_ mitigation, and *L. casei* TH14 produced a smaller independent reduction. However, combining *L. casei* TH14 with hydrolysable tannin did not produce effects beyond those of hydrolysable tannin alone, indicating an additive rather than a synergistic interaction. Overall, sweet chestnut hydrolysable tannin at 40 g/kg DM is a promising corn silage additive for mitigating in vitro CH_4_ production while preserving nutrient digestibility. Further in vivo studies are warranted to confirm these effects, including organic acid profiling, microbial enumeration, and measures of feed intake, rumen adaptation, and animal performance.

## Figures and Tables

**Table 1:** The ingredients and chemical composition of the concentrate diet used as a fermentation substrate.

Item	Concentrate Diet
Ingredients (% DM)	
Cassava chips	45.0
Corn meal	10.0
Rice bran	10.0
Soybean meal (SBM)	11.0
Dried brewers’ grains	11.0
Palm kernel meal	10.0
Minerals and vitamins ^1^	1.0
Urea	0.5
Salt	1.0
Molasses	0.5
Chemical composition	
Dry matter, %	94.18
Organic matter, %DM	93.51
Crude protein, %DM	14.01
Neutral detergent fibre, %DM	42.27
Acid detergent fibre, %DM	16.97
Gross energy (MJ/kg DM)	16.90

^1^ Mineral and vitamin quantities per kg of premix: Vitamin A, 10,000,000 IU; Vitamin D, 1,600,000 IU; Vitamin E, 70,000 IU; Fe, 50 g; Zn, 40 g; Mn, 40 g; Cu, 10 g; I, 0.5 g; Co, 0.1 g; Se, 0.1 g.

**Table 2:** Chemical composition and fermentation profile of whole-plant corn silage treated with *Lactobacillus casei* TH14 and hydrolysable tannin across ensiling days (days 0, 7, 14, and 21).

Treatment	Day	DM	OM	CP	NDF	ADF	GE	pH	NH_3_-N
T1	0	27.98	93.20	8.08	58.67	27.99	16.48	5.39 ^a^	0.71
	7	28.39 ^b^	93.29	7.83	60.99	30.44	16.65	4.06 ^b^	2.35
	14	29.12 ^b^	93.18 ^a^	8.27	59.08	27.88	17.01	4.09 ^b^	2.53
	21	29.76 ^a^	92.73 ^b^	8.18	64.21	32.36	17.08	4.12 ^b^	2.86
T2	0	29.92	93.32	8.07	59.85	28.35	16.63	5.06 ^a^	0.63
	7	28.38 ^b^	93.29	7.70	62.31	30.91	16.73	3.88 ^b^	2.05
	14	29.23 ^b^	93.12 ^a^	7.90	59.30	26.99	17.11	3.91 ^b^	1.70
	21	29.93 ^a^	93.06 ^a^	7.70	61.10	30.02	17.25	3.96 ^b^	2.07
T3	0	28.10	93.28	8.29	59.94	30.35	16.76	5.07 ^a^	0.61
	7	28.81 ^a^	92.93	8.09	63.04	32.86	17.19	4.08 ^b^	2.07
	14	30.43 ^a^	92.31 ^c^	8.12	62.72	30.86	17.14	4.04 ^b^	1.47
	21	29.66 ^b^	93.35 ^a^	8.17	62.59	32.72	17.14	3.92 ^b^	1.95
T4	0	28.02	93.22	7.94	59.52	26.02	16.93	5.07 ^a^	0.63
	7	28.81 ^a^	93.23	7.60	61.92	28.35	16.96	3.97 ^b^	1.61
	14	29.74 ^b^	93.41 ^a^	7.79	59.87	27.83	17.02	4.02 ^b^	1.20
	21	30.06 ^a^	92.36 ^b^	7.56	62.67	29.62	17.07	3.83 ^b^	3.15
T5	0	28.27	93.20	7.85	59.84	26.40	17.09	5.08 ^a^	0.54
	7	29.14 ^a^	93.15	7.47	62.26	28.71	17.08	3.89 ^b^	1.47
	14	31.04 ^a^	92.88 ^b^	7.57	59.75	25.52	17.14	4.08 ^b^	0.87
	21	30.45 ^a^	92.90 ^a^	7.75	64.53	30.52	17.17	3.96 ^b^	1.06
T6	0	28.32	93.03	8.02	62.15	28.49	16.97	4.61 ^a^	0.58
	7	29.41 ^a^	92.97	7.69	64.54	31.43	16.98	4.10 ^b^	1.73
	14	30.82 ^a^	92.78 ^b^	7.83	63.59	28.20	17.05	4.11 ^b^	1.44
	21	29.96 ^a^	92.66 ^b^	8.05	66.41	33.11	17.09	4.00 ^b^	1.47
T7	0	28.17	93.29	8.05	62.53	30.52	17.14	5.06 ^a^	0.63
	7	29.30 ^a^	93.21	7.74	64.73	32.90	17.11	4.17 ^b^	1.67
	14	30.52 ^a^	92.56 ^b^	7.83	63.84	27.75	17.18	4.20 ^b^	1.20
	21	30.37 ^a^	92.78 ^b^	8.09	64.55	31.42	17.23	4.16 ^b^	1.33
SEM		0.18	0.10	0.12	1.60	0.68	0.12	0.05	0.30
Comparison									
Treatment									
T1		28.82	93.10	8.09	60.74	29.66 ^b^	16.81 ^b^	4.42 ^a^	2.11 ^a^
T2		28.87	93.20	7.84	60.64	29.06 ^b^	16.93 ^a^	4.20 ^b^	1.61 ^ab^
T3		29.25	92.97	8.17	62.07	31.69 ^a^	17.06 ^a^	4.28 ^b^	1.52 ^ab^
T4		29.16	93.06	7.72	60.99	27.95 ^c^	16.99 ^a^	4.22 ^b^	1.65 ^ab^
T5		29.73	93.03	7.66	61.60	27.78 ^c^	17.12 ^a^	4.25 ^b^	0.98 ^b^
T6		29.63	92.86	7.90	64.17	30.30 ^a^	17.03 ^a^	4.20 ^b^	1.30 ^b^
T7		29.60	92.96	7.93	63.91	30.64 ^a^	17.16 ^a^	4.40 ^a^	1.20 ^b^
Day									
Day 0		28.10 ^C^	93.21 ^A^	8.04	60.36	28.30 ^B^	16.86 ^B^	5.05 ^A^	0.61 ^C^
Day 7		28.89 ^B^	93.15 ^A^	7.73	62.83	30.79 ^A^	16.96 ^B^	4.02 ^B^	1.84 ^A^
Day 14		30.13 ^A^	92.89 ^B^	7.90	61.16	27.86 ^B^	17.09 ^A^	4.06 ^B^	1.48 ^B^
Day 21		30.03 ^A^	92.84 ^B^	7.93	63.72	31.39 ^A^	17.15 ^A^	3.99 ^B^	1.98 ^A^
P_trt		0.6468	0.5434	0.3130	0.5794	<0.01	<0.01	<0.01	<0.01
P_day		<0.01	<0.01	0.0914	0.0797	<0.01	<0.01	<0.01	<0.01
P Trt × day		<0.01	<0.01	0.6178	0.9861	<0.01	0.3587	<0.01	0.0933

T1 = Control (no additive); T2 = Molasses (20 g/kg fresh weight); T3 = Molasses + *Lactobacillus casei* TH14 (10^5^ cfu/g FM); T4 = Molasses + hydrolysable tannin (HT) at 20 g/kg DM (Low HT); T5 = Molasses + HT at 40 g/kg DM (High HT); T6 = Molasses + *L. casei* TH14 + Low HT; T7 = Molasses + *L. casei* TH14 + High HT. DM = dry matter (%); OM = organic matter (% DM); CP = crude protein (% DM); NDF = neutral detergent fibre (% DM); ADF = acid detergent fibre (% DM); GE = gross energy (MJ/kg DM); NH_3_-N = ammonia nitrogen (mg/dL); FM = fresh matter; SEM = standard error of the mean. ^a–c^ Means bearing different lowercase superscripts differ among treatments (*p* < 0.05). ^A–C^ Means bearing different uppercase superscripts differ among ensiling days (*p* < 0.05); for variables with a significant treatment × day interaction, day comparisons were made within each treatment. P_trt, P_day, and P Trt × day = *p*-values for the treatment effect, day effect, and treatment × day interaction, respectively.

**Table 3:** Gas production kinetics and cumulative gas production of corn silages treated with *Lactobacillus casei* TH14 and hydrolysable tannin incubated with buffered rumen fluid.

Treatment		Gas Kinetics (mL/0.5 g)			Cumulative Gas (mL/0.5 g DM Basis)			
		*b*	c	*L*	12 h	24 h	48 h	96 h
T1	Control	83.55 ^b^	0.038	0.75	28.42 ^a^	48.00 ^a^	68.31 ^b^	80.44 ^b^
T2	Molasses	82.95 ^b^	0.026	0.50	21.36 ^b^	37.79 ^b^	58.62 ^b^	75.85 ^b^
T3	Molasses + *L. casei* TH14	87.90 ^b^	0.034	0.80	28.13 ^a^	48.36 ^a^	70.59 ^a^	84.58 ^b^
T4	Molasses + Low HT	109.95 ^a^	0.037	1.45	35.89 ^a^	62.66 ^a^	90.65 ^a^	106.72 ^a^
T5	Molasses + High HT	98.40 ^a^	0.034	1.10	30.45 ^a^	53.25 ^a^	78.46 ^a^	94.51 ^a^
T6	Molasses + *L. casei* TH14 + Low HT	80.35 ^b^	0.032	0.60	24.61 ^a^	42.42 ^b^	62.78 ^b^	76.58 ^b^
T7	Molasses + *L. casei* TH14 + High HT	102.00 ^a^	0.032	1.55	29.00 ^a^	52.30 ^a^	78.96 ^a^	97.05 ^a^
SEM		3.84	0.003	0.31	2.07	3.19	3.61	3.57
*p*-value		<0.01	0.3516	0.23	<0.05	<0.05	<0.01	<0.01
Orthogonal Contrast								
Contrast 1	Control	83.55 ^b^	0.038	0.75	28.42	48.00	68.31	80.44
	Molasses	93.59 ^a^	0.033	1.00	28.24	49.46	73.34	89.21
Contrast 2	Molasses	82.95	0.026	0.50	21.36	37.79	58.62	75.85
	Molasses + *L. casei* TH14	87.90	0.034	0.80	28.13	48.36	70.59	84.58
Contrast 3	without HT	85.43 ^b^	0.030	0.65	24.74 ^b^	43.07 ^b^	64.61 ^b^	80.22 ^b^
	with HT	97.68 ^a^	0.034	1.18	29.99 ^a^	52.66 ^a^	77.71 ^a^	93.71 ^a^
Contrast 4	HT Low	95.15	0.035	1.02	30.25	52.54	76.72	91.65
	HT High	100.20	0.033	1.33	29.73	52.77	78.71	95.78

T1–T7: see Table 2 footnote for treatment descriptions. *b* = maximum potential gas production (mL/0.5 g DM); c = fractional rate constant of gas production (h^−1^); *L* = lag phase (h); SEM = standard error of the mean. ^a,b^ Means within the same column bearing different superscripts differ significantly (*p* < 0.05). Planned comparisons: Contrast 1 = Control vs. Molasses-based treatments; Contrast 2 = Molasses vs. Molasses + *L. casei* TH14; Contrast 3 = without HT vs. with HT; Contrast 4 = Low HT vs. High HT.

**Table 4:** Ruminal pH, ammonia nitrogen (NH_3_-N) concentration, methane (CH_4_) production, and protozoa population of corn silages treated with *Lactobacillus casei* TH14 and hydrolysable tannin at 24 and 48 h of in vitro incubation.

Treatment		pH		NH_3_-N (mg/dL)		Methane (mL/1 g Dry Matter Substrate)		Protozoa (×10^6^ cell/mL)	
		24 h	48 h	24 h	48 h	24 h	48 h	24 h	48 h
T1	Control	6.79	6.73	12.70 ^a^	17.41	4.90 ^a^	11.70 ^a^	8.00	8.00
T2	Molasses	6.76	6.76	13.40 ^a^	17.26	5.50 ^a^	9.95 ^a^	6.00	7.00
T3	Molasses + *L. casei* TH14	6.83	6.76	12.71 ^a^	17.46	4.80 ^a^	8.55 ^b^	5.00	6.00
T4	Molasses + Low HT	6.80	6.77	11.42 ^a^	17.30	3.65 ^b^	6.60 ^b^	5.00	4.00
T5	Molasses + High HT	6.86	6.90	10.43 ^b^	17.36	2.45 ^c^	5.70 ^c^	3.00	3.00
T6	Molasses + *L. casei* TH14 + Low HT	6.85	6.92	11.21 ^a^	17.23	4.05 ^b^	6.35 ^b^	5.00	6.00
T7	Molasses + *L. casei* TH14 + High HT	6.78	6.83	11.43 ^a^	16.12	2.70 ^c^	5.20 ^c^	2.00	4.00
SEM		0.03	0.05	0.41	0.36	0.20	0.44	1.90	2.20
*p*-value		0.3774	0.1680	<0.05	0.2600	<0.01	<0.01	0.4406	0.6620
Orthogonal Contrast									
Contrast 1	Control	6.79	6.73	12.70	17.41	4.90 ^a^	11.70 ^a^	8.00	8.00
	Molasses	6.81	6.82	11.77	17.12	3.86 ^b^	7.06 ^b^	4.30	5.00
Contrast 2	Molasses	6.76	6.76	13.40	17.26	5.50 ^a^	9.95	6.00	7.00
	Molasses + *L. casei* TH14	6.83	6.76	12.71	17.46	4.80 ^b^	8.55	5.00	6.00
Contrast 3	without HT	6.80	6.76	13.06 ^a^	17.36	5.15 ^a^	9.25 ^a^	5.50	6.50
	with HT	6.82	6.85	11.12 ^b^	17.00	3.21 ^b^	5.96 ^b^	3.80	4.20
Contrast 4	HT Low	6.83	6.84	11.31	17.27	3.85 ^a^	6.47	5.00	5.00
	HT High	6.82	6.87	10.93	16.74	2.58 ^b^	5.45	2.50	3.50

T1–T7: see Table 2 footnote for treatment descriptions. NH_3_-N = ammonia nitrogen; CH_4_ = methane; SEM = standard error of the mean. ^a–c^ Means within the same column bearing different superscripts differ significantly (*p* < 0.05). Planned comparisons: Contrast 1 = Control vs. Molasses-based treatments; Contrast 2 = Molasses vs. Molasses + *L. casei* TH14; Contrast 3 = without HT vs. with HT; Contrast 4 = Low HT vs. High HT.

**Table 5:** In vitro dry matter degradability (IVDMD), organic matter degradability (IVOMD), neutral detergent fibre degradability (IVNDF), and true degradability (IVTD) of corn silages treated with *Lactobacillus casei* TH14 and hydrolysable tannin at 24 and 48 h of incubation.

Treatment		IVDMD (% DM)		IVOMD (% DM)		IVNDF (% DM)		IVTD (% DM)	
		24 h	48 h	24 h	48 h	24 h	48 h	24 h	48 h
T1	Control	55.00	63.68	57.86	66.43	71.00	79.40	83.24	88.09
T2	Molasses	58.11	64.04	61.15	66.79	74.59	80.40	85.79	89.04
T3	Molasses + *L. casei* TH14	56.61	63.77	58.95	65.84	73.00	80.00	84.66	88.63
T4	Molasses + Low HT	58.04	64.53	61.31	67.45	73.99	81.00	85.21	89.20
T5	Molasses + High HT	54.93	64.52	57.57	67.32	71.60	80.49	83.53	88.69
T6	Molasses + *L. casei* TH14 + Low HT	55.37	63.15	57.48	65.45	71.01	78.00	82.86	87.00
T7	Molasses + *L. casei* TH14 + High HT	53.29	64.20	55.50	66.27	70.01	79.01	82.61	87.83
SEM		0.97	1.22	1.12	1.00	1.17	1.15	0.67	0.66
*p*-value		0.0689	0.9787	0.0627	0.7680	0.1704	0.5987	0.0687	0.3456
Orthogonal Contrast									
Contrast 1	Control	55.00	63.68	57.86	66.43	71.00	79.40	83.24	88.09
	Molasses	56.06	64.03	58.66	66.52	72.37	79.82	84.11	88.40
Contrast 2	Molasses	58.11	64.04	61.15	66.79	74.59	80.40	85.79	89.04
	Molasses + *L. casei* TH14	56.61	63.77	58.95	65.84	73.00	80.00	84.66	88.63
Contrast 3	without HT	57.36	63.91	60.05	66.32	73.80	80.20	85.23 ^a^	88.84
	with HT	55.41	64.10	57.97	66.62	71.65	79.62	83.55 ^b^	88.18
Contrast 4	HT Low	56.71 ^a^	63.84	59.39 ^a^	66.45	72.50	79.50	84.04	88.10
	HT High	54.11 ^b^	64.36	56.54 ^b^	66.79	70.80	79.75	83.07	88.26

T1–T7: see Table 2 footnote for treatment descriptions. IVDMD = in vitro dry matter degradability; IVOMD = in vitro organic matter degradability; IVNDF = in vitro neutral detergent fibre degradability; IVTD = in vitro true degradability; SEM = standard error of the mean. ^a,b^ Means within the same column bearing different superscripts differ significantly (*p* < 0.05). Planned comparisons: Contrast 1 = Control vs. Molasses-based treatments; Contrast 2 = Molasses vs. Molasses + *L. casei* TH14; Contrast 3 = without HT vs. with HT; Contrast 4 = Low HT vs. High HT.

**Table 6:** Volatile fatty acid (VFA) concentrations of corn silages treated with *Lactobacillus casei* TH14 and hydrolysable tannin at 24 and 48 h of in vitro incubation.

Treatment		Total VFAs (mmol/L)		Acetate (C2) (mol/100 mol)		Propionate (C3) (mol/100 mol)		Butyrate (C4) (mol/100 mol)		C2:C3	
		24 h	48 h	24 h	48 h	24 h	48 h	24 h	48 h	24 h	48 h
T1	Control	44.01 ^a^	56.11 ^a^	73.54	73.25 ^a^	15.04 ^b^	14.79 ^c^	11.41 ^a^	11.96 ^a^	4.89 ^a^	4.96 ^a^
T2	Molasses	50.60 ^a^	64.06 ^a^	70.38	71.97 ^b^	16.77 ^a^	17.00 ^b^	12.85 ^a^	11.03 ^b^	4.20 ^b^	4.23 ^b^
T3	Molasses + *L. casei* TH14	53.06 ^a^	67.17 ^a^	70.41	70.07 ^d^	16.91 ^a^	19.05 ^a^	12.68 ^a^	10.88 ^b^	4.17 ^b^	3.68 ^c^
T4	Molasses + Low HT	45.13 ^a^	57.12 ^a^	68.86	72.31 ^b^	18.04 ^a^	16.54 ^b^	13.10 ^a^	11.15 ^b^	3.82 ^b^	4.37 ^b^
T5	Molasses + High HT	41.33 ^b^	52.31 ^b^	69.50	72.12 ^b^	18.29 ^a^	16.87 ^b^	12.22 ^a^	11.02 ^b^	3.81 ^b^	4.28 ^b^
T6	Molasses + *L. casei* TH14 + Low HT	47.94 ^a^	60.69 ^a^	75.69	70.50 ^c^	18.16 ^a^	18.42 ^a^	11.14 ^b^	11.07 ^b^	3.90 ^b^	3.83 ^c^
T7	Molasses + *L. casei* TH14 + High HT	42.69 ^b^	54.03 ^a^	70.29	70.88 ^c^	17.12 ^a^	18.21 ^a^	12.58 ^a^	10.91 ^b^	4.11 ^b^	3.89 ^c^
SEM		1.74	2.53	2.17	0.13	0.28	0.17	0.31	0.05	0.08	0.07
*p*-value		<0.05	<0.05	0.37	<0.01	<0.01	<0.01	<0.05	<0.01	<0.01	<0.01
Orthogonal Contrast											
Contrast 1	Control	44.01	56.11	73.54	73.25 ^a^	15.04 ^b^	14.79 ^b^	11.41 ^b^	11.96 ^a^	4.89 ^a^	4.96 ^a^
	Molasses	46.79	59.23	70.85	71.31 ^b^	17.55 ^a^	17.68 ^a^	12.43 ^a^	11.01 ^b^	4.00 ^b^	4.05 ^b^
Contrast 2	Molasses	50.60	64.06	70.38	71.97 ^a^	16.77	17.00 ^b^	12.85	11.03	4.20	4.23 ^a^
	Molasses + *L. casei* TH14	53.06	67.17	70.41	70.07 ^b^	16.91	19.05 ^a^	12.68	10.88	4.17	3.68 ^b^
Contrast 3	without HT	51.83 ^a^	65.62 ^a^	70.39	71.02 ^b^	16.84 ^b^	18.02 ^a^	12.77	10.96	4.18 ^a^	3.96
	with HT	44.27 ^b^	56.04 ^b^	71.09	71.45 ^a^	17.90 ^a^	17.51 ^b^	12.26	11.04	3.91 ^b^	4.09
Contrast 4	HT Low	46.53 ^a^	58.90	72.27	71.41	18.10	17.48	12.12	11.11 ^a^	3.86	4.10
	HT High	42.01 ^b^	53.17	69.90	71.50	17.70	17.54	12.40	10.96 ^b^	3.96	4.08

T1–T7: see Table 2 footnote for treatment descriptions. VFAs = volatile fatty acids; Total VFAs expressed as mmol/L; individual VFAs as mol/100 mol total VFA. C2:C3 = acetate-to-propionate molar ratio. SEM = standard error of the mean. ^a–d^ Means within the same column bearing different superscripts differ significantly (*p* < 0.05). Planned comparisons: Contrast 1 = Control vs. Molasses-based treatments; Contrast 2 = Molasses vs. Molasses + *L. casei* TH14; Contrast 3 = without HT vs. with HT; Contrast 4 = Low HT vs. High HT.

## Data Availability

The data presented in this study are available on request from the corresponding author.
